# Gene expression regulation by CDK12: a versatile kinase in cancer with functions beyond CTD phosphorylation

**DOI:** 10.1038/s12276-020-0442-9

**Published:** 2020-05-25

**Authors:** Seung Hyuk Choi, Seongjae Kim, Katherine A. Jones

**Affiliations:** 1 Regulatory Biology Laboratory, The Salk Institute for Biological Studies, La Jolla, CA 92037 USA; 2 Molecular Neurobiology Laboratory, The Salk Institute for Biological Studies, La Jolla, CA 92037 USA

**Keywords:** Gene regulation, Gene expression

## Abstract

Cyclin-dependent kinases (CDKs) play critical roles in cell cycle progression and gene expression regulation. In human cancer, transcription-associated CDKs can activate oncogenic gene expression programs, whereas cell cycle-regulatory CDKs mainly induce uncontrolled proliferation. Cyclin-dependent kinase 12 (CDK12) belongs to the CDK family of serine/threonine kinases and has been recently found to have multiple roles in gene expression regulation and tumorigenesis. Originally, CDK12 was thought to be one of the transcription-associated CDKs, acting with its cyclin partner Cyclin K to promote the phosphorylation of the C-terminal domain (CTD) of RNA polymerase II and induce transcription elongation. However, recent studies have demonstrated that CDK12 also controls multiple gene expression processes, including transcription termination, mRNA splicing, and translation. Most importantly, CDK12 mutations are frequently found in human tumors. Loss of CDK12 function causes defective expression of DNA damage response (DDR) genes, which eventually results in genome instability, a hallmark of human cancer. Here, we discuss the diverse roles of CDK12 in gene expression regulation and human cancer, focusing on newly identified CDK12 kinase functions in cellular processes and highlighting CDK12 as a promising therapeutic target for human cancer treatment.

## Cancer: Phosphorylating enzyme may provide therapeutic target

Better understanding of the roles played by a protein kinase, an enzyme that adds phosphate groups to other molecules, in healthy and diseased states may help scientists identify novel cancer treatments. Cyclin-dependent kinases (CDKs) are a family of protein kinases crucial to cell cycling and gene expression. CDK12 can activate and modulate cancer-related gene expression, but, according to a review by Seung Hyuk Choi and colleagues at the Salk Institute for Biological Studies in La Jolla, USA, further investigations into its exact functioning and control mechanisms are required. CDK12 mutations are frequently found in aggressive breast and ovarian cancers, while loss of CDK12 function results in abnormal expression of DNA damage response genes and genome instability. CDK12 may also regulate drug resistance in cancer cells. The team suggests that therapies targeting CDK12 are worth exploring.

## Introduction

The transcriptional cyclin-dependent kinases (CDKs) CDK7, CDK8, CDK9, CDK12, and CDK13 are evolutionally conserved and share a common phosphorylation substrate, which is the RNA polymerase II (RNAPII) C-terminal domain (CTD) heptad repeat (Y_1_S_2_P_3_T_4_S_5_P_6_S_7_). Thus, they can integrate transcription with changes in phosphorylation of RNAPII CTD throughout the transcription cycle^1,2^. Phosphorylation of the Ser5 position of RNAPII CTD by CDK7, which is a part of the transcription initiation factor TFIIH, is linked to promoter-proximal transcriptional pausing, which tends to decline during the transition to active transcription elongation, accompanied by an increase in Ser2 phosphorylation mediated by CDK9 and CDK12^3–5^. Through phosphorylation of RNAPII and associated transcription factors, the nuclear RNAPII CTD kinases coordinate multiple cotranscriptional events important for gene expression, including mRNA splicing and surveillance, termination/cleavage, and the export of nascent mRNAs^6–8^. Recent evidence has shown that CDK12 plays a unique role among CTD kinases in regulating genome stability and cancer cell drug resistance^4,9^. The expression levels of CDK12 and its partner cyclin, cyclin K (CCNK), are unchanged throughout the normal cell cycle but are frequently elevated in proliferating stem cells and cancer cells. Consistent with its role in the maintenance of genome stability, CDK12 that is lost or mutated is associated with the progression and metastasis of a subset of human cancers, including serous ovarian, breast, and prostate cancers. This review addresses emerging studies to dissect the novel mechanisms of CDK12 in gene expression regulation and its roles in human cancer and consider its potential clinical applications as a biomarker and cancer therapeutic target.

## CDK12 and evolution

**Fig. 1:**
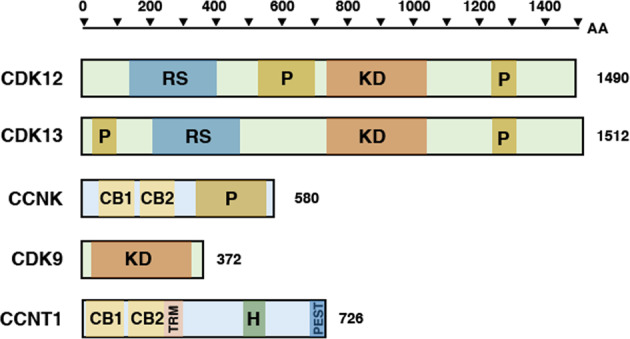
Comparison of the primary structures of CDK9, CDK12, and CDK13. KD kinase domain, RS arginine/serine-rich motifs, P proline-rich motifs, CB cyclin-box, TRM TAT/TAR recognition motif, H histidine-rich segment, and PEST PEST domain. The scale indicates the length of the amino acids (AA) and proteins.

CDK12 was first cloned from a human cDNA library to screen for Cdc2-related genes^10^. It is ubiquitously expressed and highly expressed in male and female reproductive tissues, endocrine tissues, bone marrow, spleen and lymph nodes. The gene encodes a protein of 1490 amino acids (164 kDa by prediction, 180 kDa in SDS–PAGE gel) harboring a catalytic domain in the middle of the protein (residues 719–984) and showing 42% identity with the kinase domain of human cdc2 (CDK1). The protein has multiple bipartite nuclear localization sequences (NLS) and is mainly detected in the nucleus and nuclear speckles. A prominent feature of CDK12 includes arginine/serine rich (RS) domains in the N-terminus, which are frequently found in many splicing factors; therefore, it is called a Cdc-related kinase with an arginine/serine-rich (RS) domain (CrkRS). CDK12 contains 21 RS motifs, and the presence of this RS domain is necessary for CDK12 to localize to nuclear speckles, suggesting its active role in transcription regulation. CDK12 and its most homologous kinase, CDK13 (also called CDC2L5, 1512 amino acids), are uniquely long among CDKs with distinct structural characteristics, including an RS domain and a proline-rich (P) domain, which might mediate RNA–protein or protein–protein interactions. Schematic diagrams of the structural features of CDK12, Cyclin K, and related proteins are illustrated in Fig. 1. Evolutionally, CDK12 is highly conserved from yeast and flies to humans. The fly (*Drosophila melanogaster*) CDK12 has structural features similar to those of human CDK12, but the yeast (*Saccharomyces cerevisiae*) ortholog CTK1 shows low structural similarity, with its core kinase domain mostly conserved. Another name for CDK12, CrkRS, is no longer used because several studies confirmed that CDK12 is associated with a unique cyclin, Cyclin K, a characteristic feature of a CDK complex; it is now called CDK12^3–5^. CDK13 also binds to Cyclin K and forms a separate complex^4^. The CDK12–Cyclin K and CDK13–Cyclin K complexes may share similar functions because of the similarity of their structures and associated cyclin. Cyclin K is a 70 kDa protein with a conserved cyclin box in the N-terminus and a proline-rich region in the C-terminus. Knocking down CDK12 expression results in decreased stability of Cyclin K and vice versa^4,11^, indicating that the interaction between CDK12 and Cyclin K is important for complex integrity and stability. The yeast ortholog of human CDK12/CDK13 is CTK1. The yeast kinase protein complex (CTDK-I) consists of CTK1 (kinase), CTK2 (a cyclin partner), and CTK3 (a yeast-specific subunit). In comparison, the yeast ortholog of human CDK9/Cyclin T (P-TEFb) is the Bur1/Bur2 complex.

### Activity and structure of CDK12

The different patterns of phosphorylation events in the heptad repeats of RNAPII CTD are critical for regulating the processivity of RNAPII, the transcription elongation rate, mRNA processing, and chromatin modification, because particular combinations of phosphorylation sites in of the CTD, so-called “CTD code”, can generate specific docking sites and determine the recruitment of specific regulatory factors at the phosphorylated CTD sites^12^. The CTD kinase activity of CDK12 was first reported based on an in vitro kinase assay. In this experiment, anti-CDK12 obtained from mammalian cells via immunoprecipitation were able to phosphorylate substrates, including SR-type splicing factor ASF, myelin basic protein (MBP), and a recombinant yeast CTD protein, which led to the speculation that CDK12 is a novel CTD kinase^10^. Notably, the initial purification of the CDK12 protein expressed in baculovirus-infected cells was not successful due to its insolubility. Additional in vitro kinase experiments using fly CDK12^5^ and human CDK12 from other groups^3,4^ also showed that CDK12 effectively phosphorylated the CTD, confirming that CDK12 was a bona fide CTD kinase with potential roles in transcription regulation. The in vivo kinase activity of CDK12 as a CTD kinase was also demonstrated using RNAi in both fly cells and human cells^4,5^. Knocking down CDK12 or Cyclin K resulted in a significant reduction in CTD phosphorylation, with particularly remarkable effects on Ser2 phosphorylation. Interestingly, this effect was not as profound after CDK13 knockdown, indicating that CDK12–Cyclin K is a dominant CTD kinase complex with global control of CTD phosphorylation. A more detailed study using a phosphorylated CTD peptide substrate revealed that CDK12 prefers a CTD substrate that is prephosphorylated at the Ser7 position but has low activity toward an unmodified CTD peptide. However, P-TEFb actively phosphorylated the unmodified CTD^13^, indicating that Ser7 phosphorylation is a necessary element of the kinase motif recognized by CDK12 and that other kinases must be in place to prime this phosphorylation before CDK12 properly recognizes the CTD as the preferred substrate. Usually, higher levels of Ser7 phosphorylation in CTD are found among actively transcribed genes^14,15^. For example, in yeast, Ser7 is phosphorylated early in transcription and persists at robust levels throughout the whole transcription process^16^. Because of the CDK12 preference for this substrate, it is possible that higher CDK12 activity is associated with actively transcribed genes marked by high levels of Ser7 phosphorylation. Furthermore, using an affinity purified CDK12 complex in an in vitro kinase assay, this study also demonstrated that CDK12 can phosphorylate both the Ser2 and Ser5 positions, a finding consistent with the property of the CDK9 kinase^17^. Therefore, the substrate specificity of CDK12, according to the phosphorylation pattern in the CTD, has largely remained elusive due to the different combinations of phosphorylation at multiple sites in the CTD heptad (CTD code) and the limited number of available phospho-specific antibodies that can distinguish among them. To replace the use of these CTD-phospho-specific antibodies, a new mass-spectrometry (MS)-based approach can be used to determine and map diverse phospho-sites along the entire CTD^18^. This technique is expected to reveal the exact positions and provide quantitative measurements of phosphorylation patterns, including neighboring CTD residues, to elucidate the CTD code.

The crystal structure of the CDK12/Cyclin K complex has been resolved^13^. The overall structure of CDK12 and Cyclin K in complex is generally similar to that of other CDK complexes, with similar features including the CDK–cyclin interface, an ATP-binding pocket in the core kinase domain, and kinase activation by T-loop phosphorylation. Of interest, researchers found that human CDK12/Cyclin K expressed in a baculoviral system had only basal levels of kinase activity. However, the coexpression of yeast CAK (Cdk-activating kinase) in the expression system resulted in a significant increase in CDK12 kinase activity due to phosphorylation of Thr893 by the CAK residing in the T-loop of CDK12. Therefore, Thr893 phosphorylation in the T-loop is essential for CDK12 kinase activation. Another important structural feature is the polybasic region conserved between transcription-associated CTD kinases. The polybasic cluster (^1045^KKRRRQR) in CDK12 is extended from the C-terminal kinase domain and forms a helical structure in parallel orientation to the C-terminal part of the kinase domain. It was demonstrated that the truncation of this C-terminal extension, including the polybasic motif, causes a gradual loss of kinase activity, suggesting that its presence is necessary for maintaining robust catalytic activity by blocking the free exchange of ADP to ATP in the ATP-binding pocket. From these observations, potential regulatory mechanisms for CDK12 kinase activity might be based on switching of T-loop phosphorylation or protein–protein interactions in the polybasic cluster region, which may be used for a protein docking site. Therefore, further study will be important to understand how CDK12 activity is controlled from the point of view of the potential CDK12-activating kinases or the CDK12-binding proteins via the polybasic motif.

### CDK12 as a positive transcriptional regulator

It has been generally thought that CDK12 is globally associated with transcription elongation, similar to CDK9, based on its predominant kinase activity toward the Ser2 position of the heptad repeats in the RNAPII CTD^3–5^. However, depletion of CDK12 function in human cells affects <5% of transcription and selectively affects a uniquely defined group of genes, indicating that CDK12 must invoke a gene-targeting mechanism upon transcription activation. A parallel example was shown by the transcription activation of Nrf2 target genes in flies. Nrf2 is a master transcription factor in reactive oxygen species (ROS)-induced transcription activation. Nrf2 is rapidly induced by diverse ROS stresses to bind to the promoter of a downstream target gene to control its expression. From screening using RNAi in a fly system, Drosophila CDK12 was identified as a positive regulator of the transcription program governed by Nrf2 to promote stress resistance and overall survival under oxidative stress conditions^19^. Interestingly, a defined set of genes affected by CDK12 depletion differs among species. Depletion of CDK12 in flies has mostly been shown to affect redox/antioxidant genes and housekeeping genes that control metabolic activities. In humans, CDK12 is required for the transcription of DNA damage repair and DNA damage response (DDR) genes, including *BRCA*, *ATR*, *FANCI*, and *FANCD2*, which are critical for maintaining genomic stability. However, it is possible that, to some degree, CDK12 might globally regulate transcription elongation by enhancing overall Ser2 phosphorylation at the RNAPII CTD. Different ChIP-Seq experiments using CDK12-specific antibodies have consistently reported that CDK12 has genome-wide associations with promoters, protein-coding regions, and enhancers in actively transcribed genes, with extensive RNAPII signal overlap. Intensive signals of CDK12 occupancy are often found at the transcription start site (TSS), and the strongest signals of RNAPII occupancy are mostly found at promoter regions where RNAPII pauses. In this context, the polymerase-associated factor 1 (PAF1) complex, known as a general transcription elongation factor, can play an important role in the recruitment of CDK12 to genes^20^. In this report, PAF1 knockdown in human leukemia cells globally decreased CDK12 occupancy and reduced the levels of Ser2 phosphorylation in the CTD, outcomes consistent with the results from a yeast study^21^, indicating that CDK12 can play a role in general transcription elongation. In addition, a study using a CDK12-specific chemical inhibitor provided a unique perspective on these findings. Low-dose (50 nM) treatment with the CDK12 inhibitor THZ531 particularly downregulated the expression of core DDR genes, a result identical to that after genetic CDK12 depletion. However, the high-dose treatment (200 nM) of THZ531 negatively affected the expression of superenhancer-associated genes, which was not observed after the low-dose treatment^22^. Therefore, it is possible that two different transcription mechanisms could be regulated by CDK12; that is, CDK12 might phosphorylate unknown transcription factors that are highly specific for DDR gene expression, but the superenhancer genes might be controlled by the general mechanism of transcription elongation that is mediated according to the levels of CTD phosphorylation of multiple CTD kinases, including CDK12. In a study comparing CDK13, a homolog of CDK12, the transcription of some DDR genes depended solely on CDK12, not on CDK13, although other DDR genes showed more profound transcription defects upon the dual knockdown of CDK12 and CDK13^23^. The weak coincidental frequency of differentially expressed genes upon the chemical inhibition or genetic knockdown of CDK12 or CDK13 suggests that CDK12 and CDK13 have different roles in transcription elongation and target gene selection.

### CDK12 as a posttranscriptional regulator

**Fig. 2:**
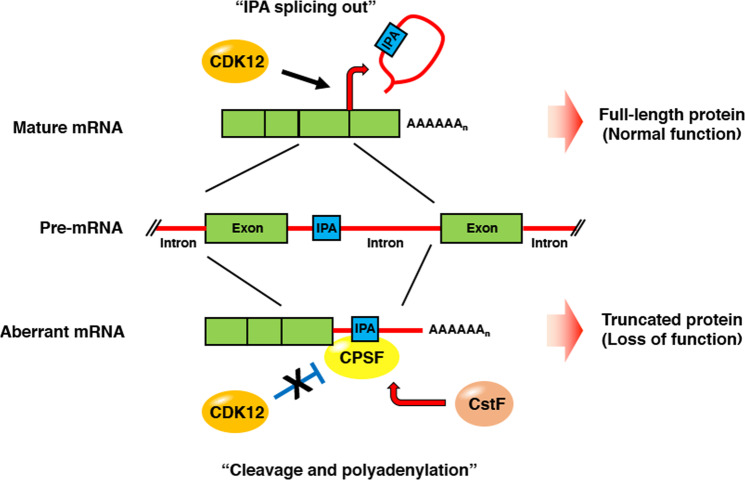
A model for CDK12 control in the maturation of mRNAs harboring intronic polyadenylation (IPA) sequences. CDK12 facilitates splicing of introns that contain IPA (blue box) and suppresses transcription to generate truncated isoforms of mRNA. Upon losing its function by mutation, CDK12 activity is decreased or delayed, and the remaining IPA-containing intron is not spliced. This event increases “cleavage and polyadenylation” by recruiting CPSFs (a cleavage and polyadenylation specificity factor, shown as a yellow circle) and CstFs (cleavage stimulating factors, shown as orange circles) to IPA, resulting in an increase in truncated isoforms.

It is conspicuous that perturbation to CTD phosphorylation by the inhibition of either CDK9 or CDK12 has little overlapping effect on gene expression, considering that both kinases have redundant phosphorylation of the Ser2 position in CTD heptad repeats. Therefore, CDK12 might play unique roles, different from those of CDK9, in gene expression regulation. Posttranscriptional regulation serves as an additional layer of gene expression control at the RNA level after RNAPII synthesizes mRNAs. CDK12 can function posttranscriptionally, for example, in the process of RNA splicing, transcription termination, and RNA export. The initial study of CDK12 suggested that CDK12 may have potential roles in RNA splicing due to the presence of multiple RS domains that are typical of many RNA-interacting and splicing factors and its subcellular location at nuclear speckles^10^. Consistent with this possibility, a protein interaction network analysis of the CDK12 protein by mass spectrometry revealed that CDK12 is associated with several RNA-binding proteins in the exon junction complex (EJC), SR splicing factors (SRSFs), and the CDC5L/PRPF19 complex. Furthermore, the N-terminal RS domains in CDK12 are required for its interaction with SRSFs, allowing CDK12 to recruit RNA cleavage and polyadenylation factors at the 3′ end for the processing of the c-FOS transcript via the RS domains^24^. Another study showed that CDK12 and CDK13 interact with SRSFs and affect the alternative splicing of the *SRSF1* gene^25^. Similarly, CDK13 was shown to regulate the alternative splicing of HIV by interacting with the splicing factor SRSF1^26,27^. Notably, a deep bioinformatic analysis of the CDK12 transcriptome showed that CDK12 can take both gene-type and cell-type-specific control of alternative splicing, known as alternative last exon (ALE) splicing^28^. In breast cancer cells, ALE splicing by CDK12 regulates the expression of DDR activator *ATM* and *DNAJB6* isoforms, which can influence tumorigenesis when CDK12 is mutated or silenced. In addition to alternative splicing, loss of CDK12 can also lead to premature termination of long transcripts with intronic polyadenylation (IPA). IPA leads to mRNA isoforms with different coding sequences or truncated transcripts that frequently result in loss of function^29^, as illustrated in Fig. 2. A recent study showed that CDK12 globally regulates the IPA of a set of genes, including DDR and homologous recombination (HR) genes, playing an essential role in the maintenance of genome stability. This study found that loss of CDK12 in human cancers globally increases IPA, resulting in a decrease in normal distal polyadenylation and the production of aberrant mRNAs^30,31^, which is a detailed mechanism by which CDK12 regulates the expression of DDR and HR genes, and CDK12 mutation potentially drives tumorigenesis. To support the oncogenic role of IPA, another study demonstrated that IPA is frequently upregulated to produce truncated mRNAs and proteins encoded by tumor suppressor genes in cancer^32^, suggesting a novel regulatory function of CDK12 to suppress the IPA of tumor suppressor genes. In conclusion, it is obvious that CDK12 and CDK13 can regulate the gene expression of a set of genes through mRNA splicing or 3′ end processing, but the precise roles and mechanisms of CDK12 in these events remain to be elucidated; specifically, the means by which kinase activity directly controls those posttranscriptional regulators requires the identification of potential phosphorylation substrates.

### Translation control by CDK12

**Fig. 3:**
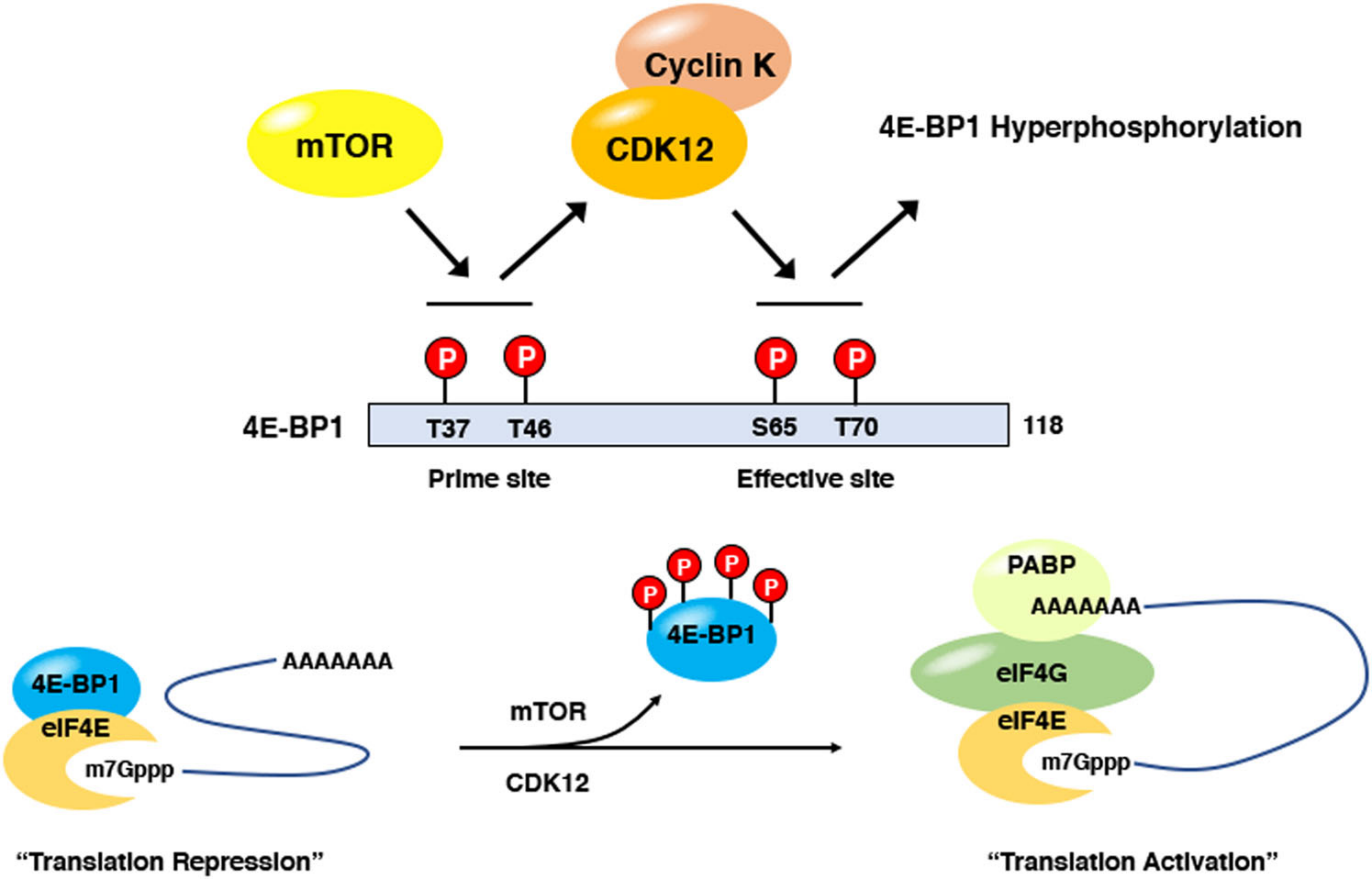
A schematic diagram of the two-step phosphorylation model for 4E-BP1 for translation control by CDK12. mTOR phosphorylation (T37 and T46 site; prime site) facilitates CDK12 phosphorylation at two consensus Ser/Thr-Pro sites (S65-P and T70-P site; effective site). Full phosphorylation events in the prime and effective sites allow 4E-BP1 to be released from the eIF4E:mRNA complex, eIF4G recruitment and PABP activation for full translational activation. When 4E-BP1 is released, the active translation complex is formed through the recruitment of translation activators (eIF4G and PABP) to the eIF4E:mRNA complex shown below.

Translational control is another layer of gene expression regulation that controls protein synthesis from a given mRNA species. In general, translation is controlled through two different ways: global control, whereby the translation of most mRNAs in the cell is affected, and mRNA-specific control, in which the translation of a specific mRNA or defined group of mRNAs is regulated without affecting the overall protein synthesis or translation efficiency of the cells. It is also thought that global control mainly occurs through the regulation of general translation factors by modifications, such as phosphorylation, whereas mRNA-specific control is mostly mediated by regulatory protein complexes that bind to specific mRNAs; these complexes can recognize particular elements that are usually encrypted in the 5′ and/or 3′ untranslated regions (UTRs) of the target mRNAs, which form specific secondary structures of these mRNAs. It is well known that several kinases play important roles in both global and mRNA-specific translational control, although the roles of CDKs in translational regulation have not been well defined. An early report showed that mitotic CDK1 can phosphorylate 4E-BP1 in a cell cycle-dependent manner^33^. In cap-dependent translation, mTOR kinase is critical for the phosphorylation of the translation repressor 4E-BP1, which is released from the mRNA–eIF4E complex, enabling translation complex assembly and further translation activation. However, the mTOR activity level is relatively low in mitosis, leading to the speculation that CDK1 can control cap-dependent translation, specifically during mitosis; thus, active CDK1 can sufficiently compensate for low mTOR activity^34,35^. Indeed, CTK1, a yeast ortholog of CDK12, plays active roles in translation regulation. CTK1 physically associates with translating ribosomes and enhances their translational accuracy through phosphorylation of the ribosomal protein Rps2, and the loss of CTK1 function causes a decrease in translation efficiency and growth defects^36^. A follow-up study in yeast extensively demonstrated that depletion of CTK1 impaired the formation of the 80S initiation complex, resulting in attenuated cap-dependent translation initiation; however, the translation of internal ribosome entry site (IRES)-containing mRNAs was not affected^37^. We recently reported that, in mammalian cells, human CDK12 can directly phosphorylate 4E-BP1 and control translation of a set of mitotic genes, as shown by a genome-wide analysis^11^. In contrast to yeast CTK1, which stimulates mRNA translation globally, our next-generation sequencing (NGS)-based ribosome-profiling analysis (Ribo-Seq) was used in our study, and it revealed that CDK12 is necessary for protein synthesis of a group of 200 selective genes. Remarkably, the majority of CDK12 target genes essentially functioned in mitotic progression. From the mechanistic view of translation control, the phosphorylation status of 4E-BP1 was dramatically altered by the depletion of CDK12 in the cells, a finding corroborated by the ability of the affinity-purified CDK12 to phosphorylate 4E-BP1 in the reconstituted in vitro kinase assay. Moreover, this kinase activity was profoundly enhanced by phosphorylation priming by the mTOR kinase complex, strongly suggesting that a certain set of mTOR-dependent genes requires CDK12 activity for efficient translation, as modeled in Fig. 3. Therefore, CDK12-depleted cells display multiple mitotic defects, including frequent severe chromosome misalignments and spindle pole detachments, followed by a significant increase in metaphase-arrested cells due to spindle assembly checkpoint activation. Notably, the CDK12 protein level is not changed throughout the cell cycle, similar to other transcriptional CDKs, but the CDK12 protein in the mitotic samples migrated more slowly on SDS–PAGE^10^, suggesting multiple phosphorylation events affected the protein during mitosis. Other evidence for mitotic-specific phosphorylation was shown by anti-CDK12 immunoprecipitates that were detected by the MPM-2 monoclonal antibody, a phospho-epitope-specific antibody that recognizes a number of phosphorylated proteins in mitosis^10^. Therefore, it is possible that the activity of CDK12 might be regulated by cell cycle-specific phosphorylation patterns, although it is not clear whether critical phosphorylation sites, such as those in the T-loop of CDK12 are regulated similarly throughout the cell cycle. Therefore, it is necessary to investigate whether the kinase activity of CDK12 or cyclin K can fluctuate through the cell cycle, and the search for potential upstream kinases for CDK12 phosphorylation at the onset of mitosis is required. Another question regarding the role of CDK12 in translation control is its predominant nuclear localization, where translation events do not usually occur. How does CDK12 cooperate with mTOR to phosphorylate 4E-BP1 and regulate translation, which is assumed to occur mainly in the cytosol? Several subunits of the mTOR complex are partly localized to the nucleus. For example, 4E-BP1 and eIF4E can move in and out of the nucleus, controlling the nuclear export of specific mRNAs^38,39^. In this respect, CDK12 may phosphorylate 4E-BP1 in the nucleus and even function cotranscriptionally to remodel the cap complex of nascent target mRNAs and facilitate transcription by phosphorylation of the RNAPII CTD. In summary, little is known about the involvement of CDKs in translation. However, studies on certain CDKs, including human CDK12 and yeast CTK1, showed that these kinases play critical roles in translation control through the phosphorylation of translation machinery components. Therefore, further investigation into CDKs is needed to understand how they affect gene expression throughout translation.

### CDK12 in tumorigenesis

The role of CDK12 in human cancer was first indicated in high-grade serous ovarian cancer (HGSOC), a disease characterized by a relatively high frequency of *BRCA* mutations, discovered by the Cancer Genome Atlas (TCGA) project. The analysis identified CDK12 as one of the recurrently mutated genes in 3% of HGSOC patients^40^. These CDK12 mutations were sporadic, but most of these mutations were either nonsense, indel, or missense mutations in the protein kinase domain, suggesting potential loss of CDK12 function in this type of cancer. We now understand that CDK12 is a universal regulator of DDR and HR gene expression, including BRCA1. Since BRCA1 defects are closely associated with susceptibility to breast cancer, ovarian cancer, and prostate cancer, CDK12 mutation has been similarly related to the susceptibility of these cancers, with a defect in the double-strand break repair mechanism mediated by DDR and HR defined as “BRCAness”^41,42^. In addition, it is well known that BRCA1-defective cancers are intrinsically sensitive to poly (ADP-ribose) polymerase (PARP) inhibition^43^. The advent of PARP inhibitor monotherapy established a novel concept^44^, termed synthetic lethality, whereby a specific defect in the cell creates another specific susceptibility to induce cell death. Therefore, the synthetic lethal relationship based on PARP1 inhibition could be applied to CDK12 mutants, similar to its application to BRCA1 mutants^9,45^, and CDK12 inhibition might provide a rationale for the use of combination therapy with a PARP inhibitor in CDK12 mutation-related cancers. Further study of CDK12 mutants in HGSOC demonstrated that most CDK12 mutations abolish the CDK12/Cyclin K interaction, resulting in the inactivation of CDK12 kinase, defects in the expression of HR genes, impairment of DNA double-strand break repair via HR, and reinforced sensitivity to the PARP inhibitor^9,46^. To support this finding, CDK12 was identified in a synthetic lethality screening, in which CDK12 depletion conferred sensitivity to PARP inhibition as a phenocopy of BRCA deficiency^45^. Interestingly, the majority of CDK12 mutations were shown to be mutually exclusive in BRCA1 and BRCA2 mutants, strongly indicating that CDK12 may act as an upstream effector in the same pathway in which BRCA1 and BRCA2 function as tumor suppressors^45^. In support of this hypothesis, CDK12 mutants drive defects in the expression of key DDR genes, including BRCA1, in ovarian cancer^46^. Therefore, we now consider CDK12 an upstream factor in the BRCA and HR gene response pathway, and CDK12 mutations, as well as BRCA mutations, can be therapeutically used to predict the sensitivity to PARP inhibitors in cancer treatment.

In contrast to the tumor suppressive role of CDK12 described above, CDK12 has been shown to have oncogenic functions. In most HER2 (ERBB2)-positive breast cancers, there is a significant correlation between CDK12 overexpression and tumor aggressiveness^47^. In addition to this observation, the CDK12 gene is frequently amplified in HER2-positive tumors^48^. The HER2 amplicon usually contains the CDK12 gene because CDK12 is a neighboring gene to HER2 in the human genome. In this circumstance, CDK12 is overexpressed by copy number amplification, and its activity is accordingly upregulated. It is intriguing that tumors with BRCA mutations are more likely to be HER2-negative^49^, implying that high activity of CDK12 in the HER2 amplicon in HER2-positive breast cancers maintains BRCA function and genome stability during tumor development. If high CDK12 activity has these functions, then how can HER2-positive breast cancer cells develop tumors without disrupting genome stability? It is possible that high CDK12 activity can upregulate DDR genes to promote rapid DNA replication^50^. In another possibility, CDK12 might serve as a key factor for the expression of important oncogenes. For example, MYC is a strong oncogene that is highly deregulated in most human cancers. It was demonstrated that chemical inhibition of CDK12 reduced tumor growth with the concurrent abrogation of MYC expression in patient-derived xenograft experiments^51,52^. In support of these observations, CDK12 is necessary for the 3′ end processing of MYC transcription^7^ and ribosome protein synthesis that induce MYC-driven anchorage-independent growth^11^. In addition, CDK12 was found to have a synthetic lethal relationship with MYC^53^ and EWS/FLI rearrangements in Ewing sarcoma^54^, indicating that CDK12 is an essential component in oncogene-driven tumorigenesis. Thus, it is possible that CDK12 can cooperate with oncogenic transcription factors, such as MYC and EWS/FLI, to drive oncogenic transcription programs by modulating RNAPII activity as a transcriptional cofactor^55^. In this scenario, CDK12 might be a promising therapeutic target for the cancers described (Table 1).

**Table 1:** Different functions of CDK12 in tumorigenesis.

Mutation type	Loss of function	Gain of function
CDK12 status	Loss of function mutation or deletion	Copy number amplification or overexpression
Affected regulatory mechanism	DNA damage response (DDR) Homologous recombination repair (HR)	Increased oncogene expression Cofactor of oncogene transcription program
Results	Genome instability	Oncogene program activation
Contribution	Onset of tumorigenesis	Progression of tumorigenesis
Cases	High-grade serous ovarian cancer (HGSOC) Metastatic castration-resistant prostate cancer (mCRPC)	HER2-positive breast cancer Reversion of BRCA mutation cancer
Potential therapeutic approach	PARP inhibitor to enhance sensitivity in chemotherapy	CDK12-specific inhibitors

### Pharmacological inhibition of CDK12 to treat cancers

CDKs are known to play significant roles in cell cycle progression and gene expression regulation, and they are highly active in human cancers. The development of CDK inhibitors is of great interest for clinical purposes, mainly for cancer treatments. Therefore, the identification of CDK inhibitors has been passionately pursued to treat cancers by preventing cancer cell proliferation. For example, the FDA approved the CDK4/6 inhibitor palbociclib for the treatment of hormone receptor-positive, HER2-negative metastatic breast cancer^56^. Although CDK inhibitors have adverse cross-reactivity due to the close similarity of the core kinase domains among CDKs, two CDK12-specific inhibitors were recently identified as having highly potent inhibition activity against a CDK12-mediated gene expression program and were shown to be potential anticancer agents. The first CDK12-specific inhibitor, THZ531, was derived from a covalent CDK7 inhibitor, THZ1^22^. This small molecule is a potent inhibitor of the kinase activity of both CDK12 (IC_50_ of 158 nM) and CDK13 (IC_50_ of 69 nM) and can also covalently target Cyc1039, which is located outside of the CDK12 kinase domain and is highly conserved between yeast and the human CDK12 family. A crystal structure study virtually demonstrated that the short region neighboring Cyc1039 forms an ATP cleft outside the canonical kinase domain of CDK12. This unique feature enables THZ531 to induce highly selective inhibition of CDK12/CDK13, compared to CDK7 or CDK9. Inhibition of CDK12 by THZ531 can induce anti-proliferation of cancer cells by inducing apoptosis (IC_50_ of 50 nM in Jurkat cells), presumably due to transcriptional stress, similar to its predecessor, THZ1. The other CDK12 inhibitor, SR-4835, was derived from structure-guided optimization of small molecules with high affinity for CDK12 and CDK13. In contrast to THZ531, the inhibitory mode of SR-4835 is ATP-competition via hydrogen bonding with the hinge region of the kinase core domain, which makes SR-4835 highly selective for CDK12 and CDK13, among kinases^23^. The pharmacological inhibition of CDK12 by both inhibitors suppresses the expression of DDR genes, leads to a global decrease in the Ser2 phosphorylation of the CTD and triggers apoptosis. Intriguingly, the SR-4835 study showed that the treatment induced less toxicity in primary cells than it did in tumor cells. The cause of this tumor-specific cytotoxicity is unclear. It is possible that CDK12 might govern some cancer-specific transcription programs. In this circumstance, cancers might depend on CDK12 activity for cell proliferation, and its inhibition may result in cancer-specific cell death.

In addition to the DDR genes involved in HR, PARP has an important function in DNA damage repair by maintaining genomic stability and cancer cell survival. As we discussed previously, PARP inhibitors have attracted intense interest because they can induce synthetic lethality in cancer cells harboring BRCA mutations, which is defined as “BRCAness”; defects in HR repair are mostly induced by BRCA1 or BRCA2 loss^57^. In turn, BRCA-deficient cells would utilize another, error-prone DNA repair pathway mediated by PARP. Therefore, synthetic lethality is achieved when cells are no longer able to repair DNA damage because of the dual inhibition of BRCA and PARP, induced either genetically or pharmacologically. However, this synthetic lethality has limitations in cases involving BRCA wild-type cancers, the genetic reversion of a BRCA mutant that mediates BRCAness in cancer to a form that restores HR activity and resistance to PARP inhibitors^58^. CDK12 inhibition may overcome this limitation by eliminating restored HR and forcing the cancer cells to acquire the BRCAness phenotype^59^. This possibility makes targeting CDK12 a compelling strategy to overcome the drawbacks of PARP inhibitors when clinically applicable.

## Conclusions and perspectives

CDK12, a RNAPII CTD kinase, is involved in multiple biological processes, not only in transcription elongation but also splicing, translation, and DDR gene regulation. Additionally, its function is often lost in advanced recurrent cancers, but its inhibition shows promising therapeutic potential in the treatment of BRCA mutant cancers or triple negative breast cancers. Although we have recently discovered CDK12 functions, its mechanisms in diverse functions have been elucidated. Since CDK12 is a protein kinase, the identification of its substrates, including non-CTD substrates, should be addressed in the search for novel functions. For example, CDK12 might target unknown transcriptional factors for phosphorylation to prevent internal polyadenylation and maintain the fidelity of DDR gene transcription. Together with identification of potential substrates, investigations into CDK12-associated factors that regulate CDK12 activity will be instrumental, as they were, for example, for endogenous inhibitors in the similar cases of HEXIM in P-TEFb. CDK12 is a uniquely long protein with several structural features; therefore, it possible that the long arm region of CDK12 can interact with critical regulatory factors involved in gene transcription or translation. The in vivo study of CDK12 is not well elucidated due to lethality by the CDK12 knockout. Therefore, conditional modulation of CDK12 loss or gain of function in a mouse model may lead to insights into the mechanism of CDK12 and its tumor suppressor or oncogenic roles in tumorigenesis in subsequent studies; these studies will broaden the understanding of the functions and molecular mechanisms of CDK12.

Since CDK12 mutations and alterations in the genome were found in various tumors, therapeutic approaches using CDK12 inhibition should be developed for cancer treatment. Recent CDK12-specific inhibitors are very useful tools to study the therapeutic application of CDK12, especially in combination therapy with PARP inhibitors to enhance drug sensitivity and promote chemotherapy. In conclusion, CDK12 inhibitors can effectively sensitize cancer to PARP inhibition, overcoming primary and acquired resistance, and clinical trials (NCT01434316) and the development of more powerful CDK12-specific inhibitors are expected to be promising.
